# Index Catalogue of the Surgeon-General’s Office

**Published:** 1887-01

**Authors:** 


INDEX CATALOGUE SURGEON GENERAL'S OFFICE, Vol. VII.—"Insignares to Leghorn." Government print, Washington, D. C. Complied by Major J. S. Billings, U. S. A.

This vclume contains the names of 14,688 authors, representing 5,987 volumes, and 12,372 pamphlets; also, 6,372 subject titles of separate books and pamphlets, and 34,903 titles of articles in periodicals (journals.)

There are some who do not comprehend the natue and object of this laborious compilation. It is simply a catalogue of authors and subjects, *iudexed* alphabetically, so as to enable one to find all the literature on a given subject, as far as represented in the medical library of the surgeon general's office. Thus, minor papers, contributed to medical journals, are not allowed to sink into oblivion, but may be found, read, and perhaps quoted, long after the writer has been forgotten. It is certainly creditable to the compiler, but of little use to any except those who write books.

